# SURGELLA: A Scoping Review of Impella‐Assisted Hemodynamic Support in High‐Risk Non‐Cardiac Surgery

**DOI:** 10.1111/aor.70219

**Published:** 2026-08-30

**Authors:** Hussein Elghazaly, Mohammed Allaf, Areeb Mian, Hanad Ahmed, Alex Gordon‐Weeks, Alexander Tindale, Vasileios Panoulas

**Affiliations:** ^1^ Barts Heart Centre, St Bartholomew's Hospital Barts Health NHS Trust London UK; ^2^ William Harvey Research Institute Queen Mary University of London London UK; ^3^ Royal Brompton and Harefield Hospitals Guy's and St Thomas' NHS Foundation Trust London UK; ^4^ Nuffield Department of Surgical Sciences, John Radcliffe Hospital University of Oxford Oxford UK; ^5^ Department of Hepato‐Pancreato‐Biliary Surgery Oxford University Hospitals NHS Foundation Trust Oxford UK; ^6^ Royal Papworth Hospital NHS Foundation Trust Cambridge UK; ^7^ Department of Medicine University of Cambridge Cambridge UK; ^8^ National Heart and Lung Institute Imperial College London London UK

**Keywords:** heart failure, Impella, mechanical circulatory support, non‐cardiac surgery, perioperative care, scoping review

## Abstract

**Background:**

Patients with advanced heart failure or severe ventricular dysfunction are often considered prohibitively high risk for non‐cardiac surgery. Impella may provide temporary perioperative hemodynamic support by augmenting cardiac output and unloading the left ventricle, but evidence for this strategy remains limited and largely case based.

**Methods:**

We conducted a scoping review of Impella use for adult high‐risk non‐cardiac surgery. MEDLINE and Embase were searched from inception to April 2026, with supplementary hand‐searching and citation tracking. Eligible reports were synthesized descriptively, with particular attention to implantation timing, operative context, device strategy, outcomes, and complications.

**Results:**

Twenty‐three publications comprising 36 unique patients were identified. Evidence consisted mainly of case reports and small series. Impella was used across bariatric, abdominal, oncological, endocrine, transplant‐related, vascular, orthopedic, and neurosurgical procedures. Clinical strategies included planned pre‐emptive support, surgery during established Impella or ECMELLA support, rescue implantation after perioperative deterioration, and combined support strategies. Operative completion was usually reported and 34 of 36 unique published patients survived to hospital discharge; however, this reflects selected published experience rather than a reliable estimate of effectiveness or safety.

**Conclusions:**

Impella‐supported non‐cardiac surgery appears technically feasible in highly selected patients treated in specialist centers. Current evidence supports feasibility, not comparative benefit or generalizable safety. Prospective multicenter data with standardized reporting are required before wider adoption can be justified.

## Graphical abstract

In selected patients with severe LV dysfunction, Impella‐supported non‐cardiac surgery may provide a temporary hemodynamic bridge to otherwise prohibitive procedures. Published evidence remains limited to a small number of highly selected cases, supporting technical feasibility rather than comparative benefit or generalizable safety. Prospective multicenter data are needed. HPB, hepatopancreatobiliary; ICH, intracranial hemorrhage; LV, left ventricular; LVSD, left ventricular systolic dysfunction; mAFP, microaxial flow pump. Created in BioRender. Elghazaly, H. (2026) https://BioRender.com/afmjb2q.

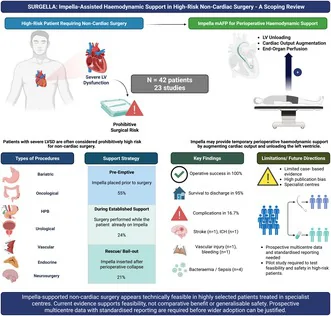

## 1 Introduction

Cardiovascular disease is common among patients assessed for non‐cardiac surgery and remains a major contributor to perioperative morbidity and mortality [1, 2]. In contemporary elective major abdominal and oncological surgery, patients with severe ventricular dysfunction or limited hemodynamic reserve are usually identified through echocardiography, cardiopulmonary exercise testing, and multidisciplinary assessment. Many are then deemed unsuitable for surgery because the anticipated perioperative risk outweighs the expected benefit.

A distinct subset of patients, however, require non‐cardiac surgery despite advanced cardiac disease. These procedures may be time‐critical or strategically necessary, including source control for sepsis, resection of malignancy where delay may compromise oncological outcomes, bariatric surgery to restore eligibility for transplantation or durable left ventricular assist device (LVAD) therapy, and selected vascular, transplant‐related, endocrine, or neurosurgical interventions. In this context, the clinical challenge extends beyond optimization of routine perioperative care, and instead centers on whether operative intervention can be safely delivered in patients who would otherwise be considered prohibitively high risk.

Such cases require careful perioperative planning and robust hemodynamic support strategies to maintain end‐organ perfusion and minimize cardiovascular decompensation, thus ensuring patient safety and optimizing clinical outcomes. Conventional management has largely relied on vasoactive drugs, fluid management, invasive monitoring, and postoperative critical care [2]. Pharmacological support alone has important limitations, including increased myocardial oxygen demand, arrhythmogenicity, and inadequate hemodynamic reserve in patients with severe ventricular dysfunction [1, 3, 4]. Temporary mechanical circulatory support (tMCS) has therefore attracted interest as a way to maintain end‐organ perfusion during surgical stress while limiting escalation of catecholamine therapy. Alternative platforms for perioperative circulatory support also exist. Veno‐arterial extracorporeal membrane oxygenation (VA‐ECMO), in particular, can be initiated rapidly in the operating theater, is comparatively inexpensive, and provides combined circulatory and respiratory support; it has been described across an expanding range of non‐cardiac and high‐risk settings, including elective procedures in patients at high perioperative cardiorespiratory risk [5]. Impella differs in offering direct left ventricular unloading through a single arterial access without an oxygenator, at the cost of isolated left‐sided support; the choice between platforms therefore depends on whether biventricular and respiratory support or targeted left ventricular unloading is the priority.

The Impella device is a percutaneous microaxial ventricular assist device that unloads the left ventricle while augmenting forward flow [6]. Its established uses are cardiogenic shock and high‐risk coronary intervention, but its perioperative use in non‐cardiac surgery has emerged as a promising adjunct in this high‐risk setting. A recent review summarized broader perioperative tMCS for cardiac assistance in thoracic surgery and highlighted the clinically useful distinction between pre‐emptive implantation and bail‐out rescue support [7]. The present review is a focused extension of that perioperative MCS field: rather than reviewing all tMCS devices in thoracic surgery, it concentrates on Impella‐supported high‐risk non‐cardiac surgery across surgical specialties.

Early reports of this approach, sometimes termed SURGELLA, suggest technical feasibility in carefully selected patients [8]. Translating this concept into clinical practice raises two clinical questions: whether tMCS can extend the boundaries of operability in patients otherwise deemed prohibitively high risk, and whether it can improve perioperative stability and outcomes in patients already considered suitable for surgery but with marginal cardiac reserve. The evidence remains limited, heterogeneous, and largely confined to case‐based experience, with no standardized selection criteria, anticoagulation protocols, reporting framework, or consensus guidance.

This review therefore summarizes the existing evidence on Impella use in high‐risk non‐cardiac surgery, including patient characteristics, procedural indications, implantation strategy, device management, outcomes, complications, whilst also identifying important gaps in current practice. By doing so, it aims to inform future research and support clinical decision‐making in this emerging area of perioperative care.

## 2 Methods

### 2.1 Search Strategy and Eligibility

A scoping review was conducted in accordance with PRISMA‐ScR principles [9]. MEDLINE and Embase were searched from inception to April 2026. Search terms combined controlled vocabulary and free‐text terms relating to Impella and percutaneous mechanical circulatory support with terms for non‐cardiac surgery and perioperative care. Terms included combinations of Impella, microaxial pump, percutaneous ventricular assist device, mechanical circulatory support, left ventricular support, non‐cardiac surgery, surgery, operation, perioperative, intraoperative, laparotomy, abdominal surgery, cholecystectomy, colectomy, nephrectomy, vascular surgery, endovascular, orthopedic surgery, and neurosurgery. Reference lists of included articles were hand‐searched, citation tracking was undertaken, and conference abstracts and gray literature were reviewed where available. The full electronic search strategy for each database is provided in File S1. The completed PRISMA‐ScR checklist is provided in File S2. The review protocol was registered with PROSPERO (CRD420261405531) and the Open Science Framework, OSF (10.17605/OSF.IO/AJHSC).

Eligible reports described adult patients receiving Impella support for perioperative hemodynamic support during non‐cardiac surgery. Case reports, case series, retrospective analyses, and conference abstracts were eligible because the scenario was expected to be rare. Reports focused exclusively on percutaneous coronary intervention, cardiogenic shock without a non‐cardiac surgical procedure, cardiac surgery, or pediatric populations were excluded.

### 2.2 Study Selection and Data Extraction

Titles and abstracts were screened independently by two authors (M.A. and A.M.), followed by full‐text review when required. Disagreements were resolved by a third reviewer (H.E.). Data extraction was performed independently by two authors (M.A. and H.A.), with discrepancies resolved by a third reviewer (H.E.). Extracted data included study design, patient characteristics, surgical indication, procedure type and urgency, Impella model, access site, implantation timing, anticoagulation strategy, support duration, perioperative outcomes, complications, escalation to other MCS, and survival to discharge where reported. Individual source‐level and case‐level extraction is provided in Table S1. To identify duplicate reporting of the same patient across abstract, case‐report, and case‐series formats, two reviewers cross‐checked all included publications by author group, center, publication date, and patient‐level characteristics (age, sex, index procedure, device model, access route, support duration, and outcome). Where the same patient appeared in more than one report, the most complete source was retained and the patient was counted once. This process identified six patients reported across overlapping publications from three centers; after de‐duplication, the 23 included publications described 36 unique patients (overlapping reports are annotated in Table 1).

**TABLE 1:** Summary of included reports.

Study	Study design	*N*	Age (years)	Sex	LVEF (%)	Procedure	Impella device	Access	Strategy	Anticoagulation	Support duration	Complications	LOS (days)	Outcome
Agrawal et al. [10]^a^	Case report	1	30	Male	NR; cardiogenic shock	Robotic sleeve gastrectomy	5.5	Axillary	Established ECMO‐Impella support	NR	Weeks	None reported	NR	Alive
Alpuerto et al. [11]	Case report	1	37	Male	NR; end‐stage heart failure	Bilateral nephrectomy	5.5	NR	Rescue after postoperative cardiac arrest	NR	Approximately 30 days as bridge to transplant	None reported	NR	Alive; heart‐kidney transplant at 30 days
Anderson et al. [12]	Case report	1	66	Female	Severe LV dysfunction	Emergency Hartmann procedure	5.5	NR	Planned preoperative	Heparin‐free purge	NR	None reported	NR	Alive
Atoui et al. [13]	Case report	1	56	Male	15%	Laparoscopic cholecystectomy	LP 2.5	Femoral	Planned same‐day preoperative support	NR	Intraoperative only	None reported	3	Alive
Bhansali et al. [14]	Case report	1	65	Male	15%	Open adrenalectomy and nephrectomy	5.0	NR	Established support as bridge to decision	NR	Weeks	None reported	NR	Alive; bridged to LVAD
Bravim et al. [15]^a^	Case report	1	55	Male	15%	Laparoscopic cholecystectomy	2.5	Femoral	Planned same‐day preoperative support	Heparin; ACT approximately 200 s	Intraoperative only	Transient troponin rise	7	Alive
Brozzi [16]	Case series	2	NR	NR	NR; cardiogenic shock	Sleeve gastrectomy (2 cases)	CP; 5.5	NR	Planned 24‐h preoperative support; established support	NR	NR	None reported	NR	Both alive; listed for heart transplantation
Chandrasekhar et al. [17]^a^	Retrospective case series	3	40.7; mean	All male	NR; cardiogenic shock, non‐ischemic cardiomyopathy	Sleeve gastrectomy (3 cases)	5.5	Axillary	Established support; mean 9.3 days	NR	Weeks	One infection	NR	All alive; bridged to LVAD
Dempsey et al. [18]	Case report	1	33	Male	Severe LV dysfunction	Open adrenalectomy (pheochromocytoma)	CP	NR	Established ECMO‐Impella support	NR	NR	ICH (fatal)	Not applicable	Died from intracranial hemorrhage
Dominedo et al. [19]	Case report	1	28	Female	20%–30%	Laparoscopic adrenalectomy (pheochromocytoma)	NR; ECMO support	NR	Impella for stabilization; removed before surgery	NR	NR	None reported	NR	Alive
Farhat et al. [20]	Case report	1	50	Male	18%	Open AAA repair	LP 5.0	Subclavian	Planned intraoperative	NR	Intraoperative only	Urinary tract infection; septic thrombophlebitis	21	Alive
Gieseke et al. [21]	Retrospective case series	6	NR	NR	NR; cardiogenic shock or heart failure	Laparoscopic cholecystectomy (6 cases)	Various	NR	Variable	NR	NR	High complication rate (cohort‐wide)	NR	20.8% 30‐day mortality in cohort
Gopalan [22]	Case report	1	60	Male	20%–25%	Sleeve gastrectomy	CP	NR	Planned preoperative	NR	36 h	None reported	4	Alive
Goswami et al. [23]	Case report	1	68	Female	10% (Takotsubo)	Renal transplant (post liver transplant)	NR; ECMELLA	NR	Rescue after post‐liver‐transplant shock	NR	Approximately 7 days	None reported	Approximately 60	Alive
Guarracini et al. [8]^a^	Case report	1	74	Male	15%	Open left hemicolectomy and colostomy	CP	Femoral	Planned same‐day preoperative support	Bicarbonate purge	Intraoperative only; 140 min	None reported	7	Alive at 6‐month follow‐up
Guarracini et al. [24]	Retrospective analysis	10	NR	NR	35% or lower	Gastrointestinal (5 cases), orthopedic (4 cases), vascular (1 case)	CP (SmartAssist)	Femoral	Planned preoperative (all patients)	NR	Intraoperative only; mean 131 min	One transfusion; no major adverse cardiovascular events	8; median	All alive; median follow‐up 227 days
He et al. [25]	Case report	1	51	Female	44%	Combined liver‐kidney transplant	5.5	Axillary	Planned intraoperative	NR	5 days	None reported	15	Alive
Lee et al. [26]	Case report	1	58	Male	22%	Robotic partial nephrectomy	CP escalated to 5.0	NR	Established support for 3 weeks	NR	Weeks	None reported	NR	Alive
Montisci et al. [27]	Case report	1	58	Male	Approximately 25%	Low anterior resection, TME and ileostomy	5.0	Axillary	Established prolonged support	Bivalirudin	More than 61 days; until death	MDR *Klebsiella* sepsis (fatal)	Not applicable	Died from sepsis
Pietrasik et al. [28]	Case report	1	47	Male	NR; EuroSCORE II 32%	EVAR (thoracoabdominal dissection)	CP	Axillary	Planned preoperative	NR	Approximately 4.5 h intraoperatively	None reported	9	Alive
Shah et al. [29]	Case report	1	66	Male	10%–16% (acute)	Orthotopic liver transplant	5.5	Axillary	Rescue after post‐reperfusion biventricular failure	NR	13 days	None reported	NR	Alive; EF recovered to 52%
Sher et al. [30]	Case series	3	26–30	All male	14%–20%	Sleeve gastrectomy (3 cases)	5.5	NR	Established support	NR	Weeks	One hepatic/pulmonary abscess	56–110	All alive; bridged to LVAD
Tagliafierro et al. [31]	Case report	1	74	Male	NR; critical left main disease	Craniotomy (hemorrhagic melanoma metastasis)	5.5	NR	Planned preoperative	Bicarbonate purge	Approximately 1 week; until post‐CABG	None reported	NR	Alive

Abbreviations: AAA, abdominal aortic aneurysm; ACT, activated clotting time; CABG, coronary artery bypass grafting; CP, Impella CP; CS, cardiogenic shock; ECMELLA, combined ECMO‐Impella support; ECMO, extracorporeal membrane oxygenation; EF, ejection fraction; ESHF, end‐stage heart failure; EVAR, endovascular aneurysm repair; GI, gastrointestinal; HF, heart failure; HKTx, heart‐kidney transplantation; HTx, heart transplantation; ICH, intracranial hemorrhage; LM, left main; LOS, length of stay; LV, left ventricular; LVAD, left ventricular assist device; LVEF, left ventricular ejection fraction; MACE, major adverse cardiovascular events; MDR, multidrug‐resistant; NICM, non‐ischemic cardiomyopathy; NR, not reported; TME, total mesorectal excision; UTI, urinary tract infection.

^a^ Reports a patient (or patients) also described in a more complete source from the same center and counted once in the synthesis: Agrawal 2024 and Chandrasekhar 2025 overlap with Sher 2024 (three unique patients, University of South Florida); Bravim 2009 overlaps with Atoui 2010; and Guarracini 2024 is included within the Guarracini 2026 cohort. Across the 23 publications there were 36 unique patients.

### 2.3 Classification of Impella Strategy

Implantation strategy was classified as pre‐emptive or prophylactic support when Impella was inserted in anticipation of hemodynamic compromise; operation during established support when surgery was performed while the patient was already receiving Impella or ECMELLA for cardiogenic shock or advanced heart failure; and bail‐out or rescue support when Impella was inserted in response to intraoperative or postoperative hemodynamic collapse. This adapts the pre‐emptive/bail‐out distinction used by Agosta et al. while preserving an additional category that is particularly relevant to Impella‐supported advanced heart failure pathways [7].

### 2.4 Outcomes and Data Synthesis

The primary outcomes of interest were feasibility and safety of Impella‐supported non‐cardiac surgery. Secondary outcomes included support duration, implantation timing, device type, access strategy, postoperative recovery, complications, escalation to other MCS, and discharge survival. Because included reports were heterogeneous and predominantly case based, meta‐analysis was not appropriate. Data were analyzed descriptively using counts, proportions, and summary measures where extractable, with emphasis on patterns of clinical use rather than pooled treatment‐effect estimates. Formal critical appraisal was not undertaken because the included evidence consisted predominantly of case reports, case series, retrospective analyses and conference abstracts; instead, evidence limitations, missingness and publication bias were considered narratively. In addition, a brief source‐level narrative appraisal was undertaken (Table S1), recording for each source its design, whether it was a peer‐reviewed full text or a conference abstract, and the completeness of reporting across baseline hemodynamics, complications, and follow‐up. Reporting was frequently incomplete, particularly for baseline hemodynamics and device settings, and a substantial proportion of sources were conference abstracts with limited methodological detail; findings should be interpreted accordingly.

## 3 Results

### 3.1 Study Selection and Study Characteristics

The PRISMA flow diagram is summarized in Figure 1. The systematic literature search identified 48 articles from electronic databases (Embase, *n* = 29; MEDLINE, *n* = 19). After removal of four duplicates, 43 records were screened. Twenty‐three publications describing 36 unique patients were included in the final synthesis; six patients reported across overlapping publications from the same centers were counted once (see Section 2 and Table 1). A summary of included reports is provided in Table 1.

**FIGURE 1:**
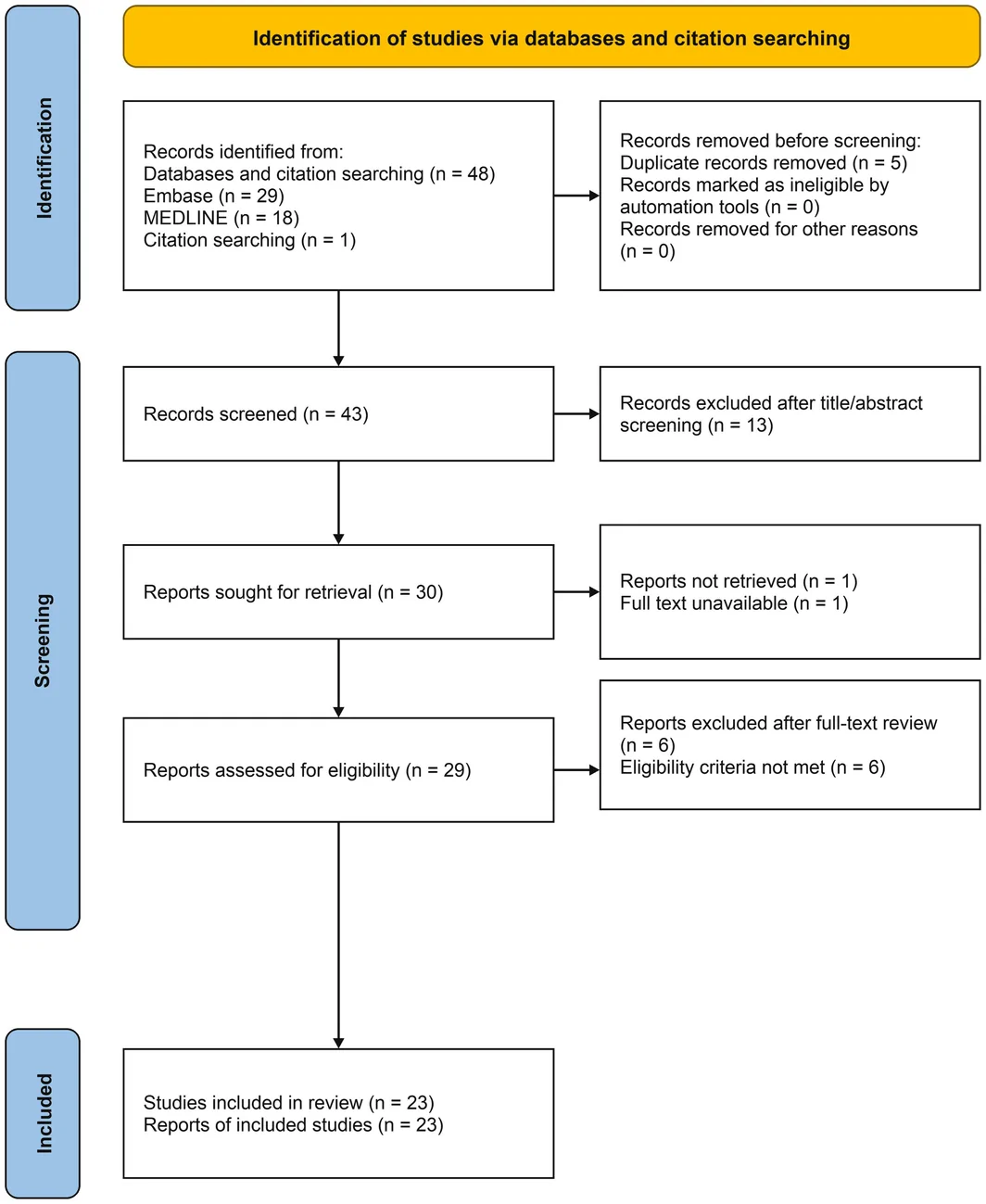
PRISMA flow diagram for study selection. The database search identified 48 records; four duplicates were removed; 43 records were screened; and 23 publications comprising 42 patients were included in the final synthesis.

The evidence base consisted predominantly of case reports (*n* = 18), with two case series and three retrospective observational studies. Publication years ranged from 2008 to 2026, with most reports published after 2020. Thirteen manuscripts originated from the United States; the remaining reports were from North America or Europe. Detailed case summaries are provided in Table S1.

### 3.2 Patient Characteristics

Baseline characteristics are summarized in Table 2. Patients were predominantly male (23 of 36; 64%); sex was unspecified in two patients. Body mass index was reported in only a minority of patients and almost entirely within the bariatric subgroup; the reported mean therefore reflects that subgroup rather than the cohort as a whole and should not be generalized. Age ranged from 28 to 75 years, with a mean age of approximately 53 years. The population was consistently high risk and was dominated by patients with advanced heart failure or severe ventricular dysfunction. Mean reported left ventricular ejection fraction was 22.56% among 25 patients with extractable values, with most cases in the 10%–25% range. One patient had moderate LV impairment (LVEF 40%–49%). Of 34 patients in whom SCAI class was reported or inferable, six (17.6%) were in SCAI D/E shock before Impella implantation. Ten patients were receiving inotropes or vasopressors, and 11 were already supported with another MCS modality, including ECMO in seven and intra‐aortic balloon pump in four.

**TABLE 2:** Baseline patient characteristics.

Total patients with extractable data	Mean (SD) or %	Comments
Male sex	64%	23 of 36 male; sex unspecified in 2
Age, years (range)	53 (14.15)	
Height (cm)	Not reported	No reported data
Weight (kg)	Not reported	
BMI	46.9 (6.3)	Reported in 4 patients across 2 studies; almost entirely the bariatric subgroup and not representative of the whole cohort
ASA	4	Reported in 1 patient (1 of 36)
HTN	47%	17 of 36
DM	36%	13 of 36
Previous CVA	2.8%	1 of 36
IHD	44%	16 of 36
CKD	28%	10 of 36
Hypercholesterolemia	36%	13 of 36
COPD	2.8%	1 of 36
Previous ACS	14%	5 of 36
Previous CABG	2.8%	1 of 36
Previous PCI	28%	10 of 36
LVEF (%)	22.56% (8.06%)	Reported in 23 patients
AF	14%	5 of 36
PAD	11%	4 of 36

Abbreviations: ACS, acute coronary syndrome; AF, atrial fibrillation; ASA, American Society of Anesthesiologists (physical status classification); BMI, body mass index; CABG, coronary artery bypass grafting; CKD, chronic kidney disease; COPD, chronic obstructive pulmonary disease; CVA, cerebrovascular accident; DM, diabetes mellitus; HTN, hypertension; IHD, ischemic heart disease; LVEF, left ventricular ejection fraction; PAD, peripheral arterial disease; PCI, percutaneous coronary intervention; SD, standard deviation.

Comorbidity reporting was inconsistent, but diabetes, hypertension, chronic kidney disease, obesity, ischemic heart disease, and previous coronary intervention were common. Several patients were active candidates for transplantation or durable LVAD therapy, emphasizing that Impella‐supported surgery was often used not simply to reduce perioperative risk, but to preserve or restore access to definitive advanced heart failure treatment.

### 3.3 Surgical Indications and Procedural Urgency

The procedures performed under Impella support were heterogeneous, with abdominal surgery forming the largest subgroup (Table 3). The most frequently reported procedures were sleeve gastrectomy (*n* = 10) and laparoscopic cholecystectomy (*n* = 8). Sleeve gastrectomy was usually performed to facilitate eligibility for transplant or durable LVAD therapy, whereas cholecystectomy was generally undertaken for definitive biliary source control in patients with advanced heart failure or cardiogenic shock.

**TABLE 3:** Spectrum of surgical interventions in Impella‐supported non‐cardiac surgery.

Category	Procedure	Number of cases
Bariatric Surgery	Laparoscopic/Robotic Sleeve Gastrectomy	6
	Gastric Bypass	1
Colorectal/Abdominal Oncological Surgery	Hemicolectomy	1
	Emergency Hartmann's Procedure	1
	Low Anterior Resection (TME) with diverting loop ileostomy	1
	Unspecified colorectal procedures	3
Hepatopancreatobiliary Surgery	Laparoscopic Cholecystectomy	7
Endocrine Surgery	Adrenalectomy	2
Urological Surgery	Nephrectomy	3
Transplant‐Related	Staged Liver‐Kidney	1
	Combined Liver‐Kidney	1
	Orthotopic Liver	1
Vascular Surgery	Open AAA repair	1
	EVAR	1
	Femoral‐Popliteal bypass	1
Orthopedic Surgery	External Tibial Fixation	1
	Knee Arthroplasty	1
	Hip Arthroplasty	2
Neurosurgery	Craniotomy for Hemorrhagic Metastatic Melanoma Mass	1

Other procedures included colorectal and oncological resections, adrenalectomy for pheochromocytoma‐associated cardiogenic shock, nephrectomy, combined or staged transplant‐related surgery, vascular interventions, orthopedic procedures, and neurosurgery.

Procedural urgency ranged from elective to emergent. Emergency indications included bowel perforation, biliary sepsis, acute vascular pathology, and hemorrhagic intracranial metastasis; urgent indications included oncological resection, transplant‐related procedures, selected vascular interventions, and orthopedic surgery where delay was considered unsafe. Elective cases were most commonly bariatric operations, as a step to facilitate transplant candidacy.

### 3.4 Timing and Strategy of Impella Implantation

Clinical strategy is summarized in Table 4, and timing of Impella insertion is summarized in Table 5. Planned pre‐emptive support included same‐day insertion immediately before induction, insertion hours to days before surgery for short stabilization, and planned intraoperative insertion before proceeding with the operative intervention. Operation during established support occurred when patients were already receiving Impella for cardiogenic shock or advanced decompensated heart failure and subsequently underwent non‐cardiac surgery while support continued. Bail‐out or rescue implantation occurred in response to perioperative collapse or hemodynamic deterioration.

**TABLE 4:** Clinically useful classification of Impella strategy.

Strategy	Operational definition	Approximate frequency	Typical clinical contexts	Representative reports	Interpretation for practice
Pre‐emptive/prophylactic support	Impella inserted before surgery, or as an initial intraoperative step, because hemodynamic compromise was anticipated.	21 of 36 (58.3%)	Planned high‐risk abdominal, oncological, transplant‐related, vascular, orthopedic, and neurosurgical procedures.	Atoui 2010; Bravim 2009; Guarracini 2024; Pietrasik 2024; He 2024; Guarracini 2026	Closest to an elective risk‐mitigation model; requires planned access, anticoagulation, anesthetic, surgical, and MCS governance.
Operation during established Impella/ECMELLA support	Non‐cardiac surgery performed while the patient was already receiving Impella or combined ECMO‐Impella support for cardiogenic shock or advanced decompensated heart failure.	6 of 36 (16.7%)	Bariatric surgery as a bridge to LVAD/transplant, oncological or renal surgery to remove barriers to advanced heart failure therapy, adrenalectomy in catecholamine shock.	Agrawal 2024; Bhansali 2020; Lee 2023; Montisci 2020; Sher 2024; Chandrasekhar 2025	Distinct from prophylaxis: the device is already part of the rescue or bridge pathway, and surgery is used to unlock recovery or definitive therapy.
Bail‐out/rescue support	Impella inserted after intraoperative or postoperative collapse, or as part of rescue escalation after acute perioperative deterioration.	9 of 36 (25.0%)	Postoperative cardiac arrest, post‐reperfusion biventricular failure, acute Takotsubo physiology, pheochromocytoma‐related cardiogenic shock.	Alpuerto 2022; Goswami 2019; Shah 2021; Dominedo 2021; Dempsey 2019	Highest‐acuity use; useful for conceptual comparison with pre‐emptive support, but published outcomes are especially vulnerable to selection and reporting bias.

**TABLE 5:** Timing and strategy of Impella implantation.

Timing pattern	Description	Examples
Same‐day pre‐operative insertion	Device inserted in cath lab or theater immediately prior to induction, then surgery proceeds	Atoui 2010; Bravim 2009; Guarracini 2024; Guarracini 2026 (cohort protocol); Pietrasik 2024
Short pre‐operative stabilization (hours–days)	Device inserted pre‐operatively with a brief stabilization period before surgery	Brozzi 2022 (24 h); Sher 2024 (Case 3—next day)
Already on Impella before surgery	Device inserted days–weeks earlier for cardiogenic shock or advanced HF; surgery later performed while supported	Lee 2023 (3 weeks); Montisci 2020 (prolonged support); Sher 2024 (Cases 1–2); Chandrasekhar 2025 (9 days); Agrawal 2024
Intra‐operative insertion	Device inserted after induction or as first operative step prior to proceeding with surgery	Farhat 2008; He 2024 (at time of transplant); some vascular/endovascular cases
Bail‐out/rescue or postoperative insertion	Device inserted after perioperative collapse or deterioration, not planned for index surgery	Alpuerto 2022; Goswami 2019; Shah 2021; Dominedo 2021; Dempsey 2019
Operation during established MCS (ECMELLA/dual support)	Surgery performed while already supported with ECMO + Impella	Dempsey 2019; Dominedo 2021; Goswami 2019

Implantation strategy was classifiable in all 36 patients. Pre‐emptive or prophylactic support accounted for 21 of 36 patients (58.3%), operation during established Impella or ECMELLA support for 6 of 36 (16.7%), and bail‐out or rescue implantation for 9 of 36 (25.0%); no case was unclassifiable by strategy. Strategy (Table 4) and the timing patterns in Table 5 are distinct descriptive axes: the timing categories are not mutually exclusive, and a single case may map to more than one. This distinction is clinically important because planned and rescue MCS represent different decision problems, risk profiles, and reporting requirements.

### 3.5 Technical Aspects of Impella Support

Access site was incompletely reported. Where stated, the femoral artery (*n* = 14) and axillary artery (*n* = 7) were the most common routes; one case used surgical cut‐down to the subclavian artery. The most common device was Impella CP (*n* = 21), followed by Impella 5.5 (*n* = 10). Four patients initially supported with Impella CP required upsizing to Impella 5.0 or 5.5. Impella 2.5 was used in two patients, and Impella was combined with ECMO (ECMELLA) in three patients.

Hemodynamic reporting was sparse. Of 42 published cases, Impella performance level was reported in only four: P8 in three patients and P7 in one. Quantitative augmentation of cardiac output was reported in four cases: two described increases of 2.0–2.5 L/min, one 3.0–5.0 L/min, and one 5.2 L/min. Anticoagulation strategy was reported in 17 cases. Heparin was used in four, sodium bicarbonate purge solutions in 12, and bivalirudin in one; heparin resistance or heparin‐induced thrombocytopenia was not explicitly described in that report.

### 3.6 Outcomes and Complications

The intended non‐cardiac procedure was completed in all 36 reported cases (100%), with no intraoperative deaths. Overall, 34 of 36 unique patients survived to hospital discharge. Two in‐hospital deaths were reported: one after catastrophic intracranial hemorrhage in a patient receiving VA‐ECMO and Impella during adrenalectomy for pheochromocytoma, and one after multidrug‐resistant *Klebsiella pneumoniae* sepsis following prolonged Impella support after oncological colorectal surgery. These outcomes should be interpreted only as the distribution of reported published cases, not as an estimate of survival after Impella‐supported non‐cardiac surgery.

Duration of support was reported in 30 patients. Eighteen patients were supported for less than 24 h, five for 1–7 days, four for 1–4 weeks, and three for more than four weeks. More prolonged support reflected use as a bridge to recovery, further optimization, or escalation to advanced heart failure therapies. Length of stay was inconsistently reported; among 12 studies reporting postoperative time to discharge, values ranged from 4 to 77 days.

Clinically significant complications were reported in seven patients (7 of 36; 19.4%); one patient may contribute more than one event. Reported events included catastrophic intracranial hemorrhage, ischemic stroke, vascular access injury requiring surgical repair, postoperative hemothorax with mediastinal hematoma, MSSA bacteremia with graft‐site thrombosis, hepatic and pulmonary abscesses, and multidrug‐resistant *Klebsiella pneumoniae* sepsis. Six patients required conversion to, or concomitant use of, VA‐ECMO. Five patients underwent or were listed for heart transplantation, and 10 required durable LVAD implantation during the index admission or follow‐up.

## 4 Discussion

Patients with severe ventricular dysfunction and limited hemodynamic reserve are frequently excluded from non‐cardiac surgery because conventional perioperative strategies relying on fluid resuscitation, vasopressors and inotropes can be insufficient and may carry clinically important trade‐offs including increased myocardial oxygen demand, arrhythmia risk, and higher perioperative morbidity [1, 2, 3, 4]. In this setting, mechanical circulatory support devices like Impella offer a distinct conceptual advantage by supporting cardiac output and ventricular unloading without significantly increasing myocardial workload; they also provide potential advantages over pharmacological agents in minimally invasive non‐cardiac surgical settings [32]. Although no direct comparator cohort exists for Impella‐supported versus conventionally managed high‐risk non‐cardiac surgery, the background risk in heart failure and severe LVSD cohorts is substantial. In a cohort of 609 735 elective non‐cardiac surgical patients, 90‐day mortality was 5.49% in patients with heart failure versus 1.22% without heart failure, rising to 8.34% with LVEF < 30% and 14.91% when LVEF < 30% was accompanied by recent heart failure symptoms [33]. Similarly, a Swedish cohort reported 30‐day, 90‐day and 1‐year mortality of 3.2%, 6.5% and 16.2% after elective surgery, and 13.7%, 22.4% and 39.3% after emergency surgery, in patients with heart failure [34].

The principal finding of this scoping review is that Impella‐supported non‐cardiac surgery remains limited, highly selected, and concentrated within specialist centers. However, within that constrained context the device has been used to facilitate a remarkably broad range of procedures that might otherwise have been considered prohibitively high‐risk. Across these reports, operative completion was generally achieved, and short‐term postoperative outcomes were often acceptable. However, these observations should be interpreted as signals of feasibility rather than proof of effectiveness, particularly given the dominance of case‐based reporting, and the high likelihood that unsuccessful or abandoned strategies are underrepresented in the published literature. This review adds to previous Artificial Organs literature from Agosta et al., which showed that perioperative temporary mechanical circulatory support can be used as pre‐emptive or bail‐out support in selected high‐risk surgical patients undergoing thoracic surgery [7]. Impella is also not the only percutaneous ventricular assist platform under active scoping‐review scrutiny. A parallel literature is emerging on pulsatile percutaneous devices such as PulseCath (iVAC2L/iVAC3L), recently the subject of its own scoping review [35], underscoring both the breadth of temporary mechanical circulatory support platforms now entering perioperative practice and the shared evidentiary immaturity, predominantly case‐based data, across the field.

The overlap is therefore conceptual rather than duplicative: Agosta et al. mapped perioperative tMCS across thoracic surgery and device types, whereas the present review isolates Impella‐supported non‐cardiac operations across abdominal, bariatric, oncological, vascular and transplant‐adjacent settings [7]. This narrower focus keeps the pre‐emptive/rescue distinction clinically useful while also exposing a third practical pattern, namely surgery undertaken during established Impella or ECMELLA support for cardiogenic shock or advanced heart failure.

Beyond the types of procedures performed, an important consideration is how patients were selected for Impella‐supported surgery. Patients selected for Impella support frequently demonstrated features of limited cardiovascular reserve, including inotrope dependence, elevated filling pressures, impaired cardiac index, and evidence of end‐organ dysfunction. In several cases, hemodynamic instability was unmasked during relatively minor interventions such as sedation or diagnostic procedures, suggesting an inability to tolerate further physiological insult. Beyond physiological considerations, the anticipated clinical benefit of surgery was a key determinant, with Impella support most often employed in scenarios where operative intervention enabled access to definitive therapies, such as transplantation, or provided potentially curative or life‐saving treatment. Collectively, these observations suggest that Impella is considered in patients with a combination of impaired hemodynamic reserve and high‐stakes surgical indication, rather than on the basis of ventricular function alone. Importantly, in many cases the role of Impella is not to reduce perioperative risk within an acceptable range, but to shift patients from inoperable to operable by augmenting hemodynamic reserve.

A notable insight from this review is that the reported cases are not random or isolated, but instead cluster into a small number of recurring clinical scenarios. These include bariatric surgery to restore eligibility for heart transplantation or durable LVAD implantation; urgent abdominal or oncological surgery in which delay risks sepsis, irreversible progression, or loss of curative opportunity [8, 27]; adrenal surgery for catecholamine‐driven cardiogenic shock; major vascular procedures in patients with life‐threatening pathology and severely impaired cardiac reserve; and transplant or transplant‐adjacent procedures in which temporary hemodynamic support may preserve access to definitive therapy. This thematic grouping highlights that Impella is most commonly employed either as a bridge to definitive therapy or to enable time‐critical surgery in patients with otherwise prohibitive cardiac risk. Importantly, these interventions are undertaken in clinical contexts where there is a clear potential for meaningful therapeutic benefits, such as restoration of transplant candidacy, control of life‐threatening disease, or delivery of curative treatments, rather than in purely palliative settings. In this context, the decision to proceed reflects a careful balance between the substantial perioperative risk and the anticipated gain from surgery, underscoring the importance of rigorous patient selection and multidisciplinary decision‐making.

From a surgical and perioperative perspective, the potential importance of this approach lies in the creation of a hemodynamic safety margin during physiologically stressful interventions. This is especially relevant in laparoscopic surgery, where induction of anesthesia, positive‐pressure ventilation, carbon dioxide insufflation, and patient positioning can substantially alter preload, afterload, pulmonary vascular loading, and cardiac output. Gallbladder pathology provides a useful example [36, 37, 38]. Acute cholecystitis in patients with advanced heart failure or critical illness may arise in a milieu of splanchnic hypoperfusion, venous congestion, systemic inflammation, and impaired gallbladder contractility, and may therefore be less responsive to conservative treatment [39, 40]. Although percutaneous cholecystostomy is often employed in high‐risk patients, current evidence supports its role primarily as a temporizing measure, with cholecystectomy remaining the definitive treatment where feasible [41]. In such cases, Impella support may help buffer the hemodynamic consequences of pneumoperitoneum and anesthesia sufficiently to allow definitive source control. That said, the available data also make clear that even when surgery is achieved, perioperative complications remain common and careful patient selection remains central.

Beyond feasibility, the implications may be particularly important in oncological surgery. For patients with resectable malignancy, cardiac inoperability may not simply increase perioperative risk but may instead eliminate the possibility of cure. Several cases in this review describe successful cancer resection under Impella support, suggesting that temporary mechanical support may preserve access to otherwise unattainable oncological treatment pathways [8, 27]. This is a clinically meaningful observation. Nevertheless, long‐term oncological endpoints are not reported, and it therefore remains unknown whether successful perioperative support translates into improvements in overall survival, recurrence‐free survival, or broader cancer‐specific outcomes. At present, the literature supports technical possibility, not oncological benefit.

### 4.1 Limitations and Future Directions

These encouraging signals must be weighed against the major limitations of the current evidence base. The literature is dominated by case reports and very small case series, with substantial heterogeneity in patient phenotype, timing of implantation, device choice, surgical indication, anesthetic strategy, anticoagulation approach, and postoperative management. Reporting of key variables is inconsistent, including baseline hemodynamics, device settings, markers of end‐organ dysfunction, criteria for device escalation or removal, and definitions of complications. Publication bias is also a serious concern, and there is very limited visibility of cases in which Impella was considered but rejected, used unsuccessfully, or associated with prohibitive complications. A somewhat more structured signal is provided by the recent single‐center retrospective pilot cohort of 10 patients with advanced heart failure undergoing planned preoperative Impella CP support, in which no 30‐day major adverse cardiac events or deaths were observed and perioperative hemodynamic stability was maintained [31]. Even so, such findings should be interpreted cautiously as they reflect carefully selected patients treated in an experienced center and cannot be assumed to represent outcomes in broader practice.

This review also has methodological limitations. Although MEDLINE and Embase were searched and citation tracking was undertaken, relevant cases may have been missed, particularly conference abstracts, non‐indexed surgical reports, device registry cases and unpublished unsuccessful uses. Although we systematically cross‐checked included reports and identified and resolved six overlapping patients (see Section 2), we cannot entirely exclude further duplication among conference abstracts that lacked sufficient identifying detail; given the small denominator, any residual overlap would modestly affect the reported proportions. In addition, because most included evidence consisted of case reports, formal estimation of complication, survival or treatment‐effect rates was not possible.

Successful integration of Impella into non‐cardiac surgical pathways also introduces important technical and logistical challenges. The presence of a percutaneous ventricular assist device requires meticulous attention to access strategy, patient positioning, draping, and intraoperative device security in order to minimize the risks of malposition, kinking, suction events, or dislodgement. In several reports, teams adapted by selecting access routes that would not conflict with the operative field, including axillary implantation for abdominal procedures, and by modifying operative technique to reduce abrupt hemodynamic perturbation [14, 15]. In laparoscopic surgery, gradual insufflation and limitation of pneumoperitoneum pressures may help attenuate reductions in venous return and increases in afterload [15]. Positioning is also highly relevant. Trendelenburg positioning, commonly required for pelvic and colorectal surgery, may increase venous return while simultaneously raising intrathoracic pressure and ventricular afterload, potentially worsening cardiac performance in severely impaired ventricles [42]. Conversely, reverse Trendelenburg, frequently used in upper gastrointestinal and biliary surgery, may reduce preload and further compromise cardiac output in patients with minimal hemodynamic reserve [43]. In this context, Impella may provide a degree of buffering against these competing physiological stresses, but its safe use depends on multidisciplinary input, with continuous communication between anesthetic, surgical, and cardiac teams to monitor device function, manage suction events, and address bleeding or thrombotic complications at the cannulation site in real‐time.

Anticoagulation represents a further unresolved challenge. The competing hazards of bleeding and thrombosis are particularly acute in non‐cardiac surgery, especially where recent device implantation, fresh operative fields, malignancy, coagulopathy, or combined organ failure coexist. Reported strategies vary considerably, from conventional systemic heparinization to bicarbonate‐based purge solutions in patients considered to be at very high bleeding risk. The increasing use of bicarbonate‐based purge solutions in contemporary practice is an important development and may reduce systemic anticoagulant exposure [44], but it should not be mistaken for a complete solution. Bleeding complications remain a major issue in patients receiving Impella support, including access‐site bleeding, procedural bleeding, and systemic hemorrhage, even if these risks appear lower in planned elective use than in emergency implantation [45, 46]. More consistent reporting of anticoagulation protocols, bleeding definitions, and thrombotic outcomes is therefore essential if meaningful perioperative guidance is to emerge.

Equally important is the question of where such care can be delivered safely. The successful use of Impella in this setting is highly dependent on institutional expertise and infrastructure. The required multidisciplinary environment, encompassing cardiologists, cardiac intensivists, anesthetists, perfusionists, cardiothoracic surgical backup, and access to extracorporeal membrane oxygenation, is currently limited to a small number of advanced centers worldwide. In addition, real‐world operational challenges, including anticoagulation management, access‐site complications, and device troubleshooting, may be difficult to manage in less experienced institutions [47]. Decision‐making should therefore extend beyond conventional “heart team” models [48] which exist in many specialized institutes. For these patients, optimal planning requires an expanded perioperative forum that includes heart failure cardiology, cardiac surgery, anesthesia, critical care, and the relevant non‐cardiac surgical specialty, with explicit discussion of indication, reversibility, operative necessity, access route, anticoagulation strategy, bailout options, and postoperative destination. That level of granular, anticipatory multidisciplinary planning is likely to be one of the true determinants of success.

From an innovation perspective, the current evidence base aligns with the early phases of the IDEAL framework, most consistent with Stage 1 (Idea) to Stage 2a (Development), characterized by case reports, small series, and exploratory application in highly selected patients. At this stage, emphasis should be placed on prospective data capture, transparent reporting, and methodological oversight, rather than widespread clinical adoption. Accordingly, the use of Impella in non‐cardiac surgery should remain confined to specialist centers operating within structured governance frameworks, where careful patient selection, multidisciplinary decision‐making, and predefined outcome reporting can support safe evaluation and iterative refinement of the technique.

Given the limitations inherent to the initial nature of existing data, advancement in this field now demands a shift towards structured prospective investigation. A pragmatic multicenter pilot study in carefully selected centers with established mechanical circulatory support expertise would be both feasible and valuable. Consecutive patients undergoing Impella‐supported non‐cardiac surgery should be enrolled prospectively, with standardized capture of preoperative hemodynamics, end‐organ function, implantation strategy, anticoagulation, device‐related complications, bleeding, need for escalation, intensive care utilization, and medium‐term survival. Such a framework would provide a reliable foundation for judging whether this strategy merely works technically in exceptional cases or whether it confers reproducible clinical benefit in defined patient groups. It would also help identify which procedures, hemodynamic phenotypes, and institutional settings are most suitable for future study.

## 5 Conclusions

Impella‐supported non‐cardiac surgery may be feasible for a small and highly selected group of patients with advanced heart failure or profound hemodynamic vulnerability who would otherwise be denied necessary operative treatment. The current literature supports technical possibility, not comparative effectiveness, generalizable safety, or best practice. Until prospective data are available, this approach should remain confined to experienced centers with structured multidisciplinary governance, clear rescue pathways, and standardized outcome reporting.

## Author Contributions

Hussein Elghazaly: conceptualization, supervision, writing (original draft and review). Mohammed Allaf: investigation, data curation, screening. Areeb Mian: investigation, screening. Hanad Ahmed: data extraction. Alex Gordon‐Weeks: writing (review and editing). Alexander Tindale: writing (review and editing). Vasileios Panoulas: conceptualization, supervision, writing (review and editing). All authors have read and approved the final version of the manuscript.

## Funding

The authors have nothing to report.

## Ethics Statement

Ethical approval was not required for this scoping review as it involved synthesis of previously published data only.

## Consent

The authors have nothing to report.

## Conflicts of Interest

The authors declare no conflicts of interest.

## Supporting information


**File S1:** Electronic search strategy.
**File S2:** PRISMA‐ScR checklist.
**Table S1:** Detailed case summaries of included reports.

## Data Availability

All data supporting the findings of this review are available within the article and its Supporting Information. Further information is available from the corresponding author upon request.
