# Supplementary material for: SURGELLA: A Scoping Review of Impella‐Assisted Hemodynamic Support in High‐Risk Non‐Cardiac Surgery
Source: Artif Organs. 2026 Aug 30;50(11):1532–45. doi: 10.1111/aor.70219 (PMC13720654; doi:10.1111/aor.70219)
Supplement: Supplementary file 1 [file AOR-50-1532-s001.docx]

**Supplementary Material**

SURGELLA - Impella-Assisted Haemodynamic Support in High-Risk Non-Cardiac Surgery: A Scoping Review

**Contents**

Supplementary File S1. Electronic search strategy

Supplementary File S2. PRISMA-ScR checklist

Supplementary Table S1. Detailed case summaries of included reports

# Supplementary File S1. Electronic Search Strategy

This supplementary file reports the reproducible electronic search strategy for the scoping review of Impella-assisted haemodynamic support in high-risk non-cardiac surgery.

## Search Overview

## Ovid MEDLINE Search Strategy

1. exp Heart-Assist Devices/ OR exp Ventricular Assist Devices/

2. (Impella* OR "microaxial pump*" OR "micro-axial pump*" OR "microaxial flow pump*" OR "micro-axial flow pump*" OR "microaxial blood pump*" OR "micro-axial blood pump*" OR "percutaneous ventricular assist device*" OR pVAD* OR "temporary mechanical circulatory support" OR tMCS OR "ventricular unloading").ti,ab,kf.

3. 1 OR 2

4. exp Surgical Procedures, Operative/ OR exp Perioperative Care/ OR exp Intraoperative Care/ OR exp Postoperative Care/ OR exp Laparoscopy/ OR exp Bariatric Surgery/ OR exp Cholecystectomy/ OR exp Colectomy/ OR exp Nephrectomy/ OR exp Transplantation/ OR exp Vascular Surgical Procedures/ OR exp Neurosurgical Procedures/ OR exp Orthopedic Procedures/

5. (surg* OR operat* OR perioperat* OR intraoperat* OR postoperat* OR "non cardiac" OR noncardiac OR non-cardiac OR laparoscop* OR laparotom* OR bariatric* OR gastrectom* OR cholecystectom* OR colectom* OR colonic OR colorectal OR nephrectom* OR adrenalectom* OR phaeochromocytoma OR pheochromocytoma OR transplant* OR vascular OR aortic OR aneurysm OR neurosurg* OR orthop?edic* OR oncolog* OR cancer OR resection* OR "source control").ti,ab,kf.

6. 4 OR 5

7. 3 AND 6

## Embase Search Strategy

1. 'heart assist device'/exp OR 'ventricular assist device'/exp OR 'percutaneous ventricular assist device'/exp

2. impella*:ti,ab,kw OR "microaxial pump*":ti,ab,kw OR "micro-axial pump*":ti,ab,kw OR "microaxial flow pump*":ti,ab,kw OR "micro-axial flow pump*":ti,ab,kw OR "microaxial blood pump*":ti,ab,kw OR "micro-axial blood pump*":ti,ab,kw OR "percutaneous ventricular assist device*":ti,ab,kw OR pVAD*:ti,ab,kw OR "temporary mechanical circulatory support":ti,ab,kw OR tMCS:ti,ab,kw OR "ventricular unloading":ti,ab,kw

3. 1 OR 2

4. 'surgery'/exp OR 'perioperative care'/exp OR 'intraoperative care'/exp OR 'postoperative care'/exp OR 'laparoscopy'/exp OR 'bariatric surgery'/exp OR 'cholecystectomy'/exp OR 'colectomy'/exp OR 'nephrectomy'/exp OR 'transplantation'/exp OR 'vascular surgery'/exp OR 'neurosurgery'/exp OR 'orthopedic surgery'/exp

5. surg*:ti,ab,kw OR operat*:ti,ab,kw OR perioperat*:ti,ab,kw OR intraoperat*:ti,ab,kw OR postoperat*:ti,ab,kw OR "non cardiac":ti,ab,kw OR noncardiac:ti,ab,kw OR "non-cardiac":ti,ab,kw OR laparoscop*:ti,ab,kw OR laparotom*:ti,ab,kw OR bariatric*:ti,ab,kw OR gastrectom*:ti,ab,kw OR cholecystectom*:ti,ab,kw OR colectom*:ti,ab,kw OR colonic:ti,ab,kw OR colorectal:ti,ab,kw OR nephrectom*:ti,ab,kw OR adrenalectom*:ti,ab,kw OR phaeochromocytoma:ti,ab,kw OR pheochromocytoma:ti,ab,kw OR transplant*:ti,ab,kw OR vascular:ti,ab,kw OR aortic:ti,ab,kw OR aneurysm:ti,ab,kw OR neurosurg*:ti,ab,kw OR orthop?edic*:ti,ab,kw OR oncolog*:ti,ab,kw OR cancer:ti,ab,kw OR resection*:ti,ab,kw OR "source control":ti,ab,kw

6. 4 OR 5

7. 3 AND 6

## Screening Application

Records retrieved by the database searches were de-duplicated and screened by title/abstract, then full text where required. Eligible reports described adult patients receiving Impella support for perioperative haemodynamic support during non-cardiac surgery. Reports focused exclusively on percutaneous coronary intervention, cardiac surgery without a non-cardiac surgical procedure, paediatric patients, or non-Impella support were excluded during screening/full-text review.

## Notes

No exclusion line using NOT cardiac surgery was applied in the proposed strategy, because relevant perioperative reports may mention cardiac surgery, LVAD, transplantation, cardiogenic shock, or mechanical circulatory support even when the index procedure is non-cardiac. Exclusions should therefore be applied during screening rather than by an aggressive search filter.

**Supplementary File S2. PRISMA-ScR Checklist**

| **Section** | **Item** | **Paraphrased requirement** | **Reported location** | **Status / action** |
| --- | --- | --- | --- | --- |
| Title | 1 | Identify the manuscript as a scoping review. | Title page, p. 1 | Reported |
| Abstract | 2 | Provide a structured summary of background, methods, results and conclusions. | Abstract, p. 2 | Reported |
| Introduction | 3 | Explain the rationale for the review. | Introduction, pp. 3-4 | Reported |
| Introduction | 4 | State the review objective or question. | End of Introduction, p. 4 | Reported |
| Methods | 5 | Indicate whether a protocol or registration exists. | Methods, p. 4 | Reported: the protocol was registered with PROSPERO and OSF. |
| Methods | 6 | Describe eligibility criteria for reports/sources. | Methods, p. 4 | Reported |
| Methods | 7 | Describe information sources and the date searched. | Methods, p. 4; Supplementary File S1 | Reported |
| Methods | 8 | Present the search strategy with sufficient detail. | Methods, p. 4; Supplementary File S1 | Reported |
| Methods | 9 | Describe source-selection procedures. | Study selection and data extraction, p. 4 | Reported |
| Methods | 10 | Describe data-charting/extraction process. | Study selection and data extraction, p. 4 | Reported |
| Methods | 11 | List and define data items extracted. | Study selection and data extraction, p. 4 | Reported |
| Methods | 12 | If critical appraisal was performed, describe the method. | Outcomes and data synthesis, p. 4 | A formal appraisal instrument was not used; a narrative source-level appraisal (design, source type, completeness of reporting) is provided in Supplementary Table S1 and summarised in the Methods. |
| Methods | 13 | Describe how results were summarised or synthesised. | Outcomes and data synthesis, p. 4 | Reported |
| Results | 14 | Report numbers screened, assessed and included. | Results, p. 5; Figure 1, p. 24 | Reported |
| Results | 15 | Present characteristics of included evidence sources. | Results, p. 5; Table 1, pp. 17-18; Supplementary Table S1 | Reported |
| Results | 16 | If critical appraisal was performed, present source-level appraisal results. | Supplementary Table S1; Results | A formal appraisal instrument was not used; a narrative source-level appraisal (design, source type, completeness of reporting) is provided in Supplementary Table S1 and summarised in the Methods. |
| Results | 17 | Present results for individual evidence sources. | Table 1, pp. 17-18; Supplementary Table S1 | Reported |
| Results | 18 | Summarise the evidence in relation to the review objectives. | Results, pp. 5-7; Tables 2-5, pp. 19-22 | Reported |
| Discussion | 19 | Summarise main findings and their relevance. | Discussion, pp. 8-9 | Reported |
| Discussion | 20 | Discuss limitations of the review process and evidence base. | Limitations and Future Directions, pp. 9-10 | Reported |
| Discussion | 21 | Provide conclusions and implications. | Conclusion, p. 11 | Reported |
| Funding | 22 | Describe funding for the review and, where assessed, funding of included sources. | Funding statement, p. 12 | Reported: no funding was received for this review. |

Source: Tricco AC, Lillie E, Zarin W, et al. PRISMA Extension for Scoping Reviews (PRISMA-ScR): checklist and explanation. Ann Intern Med. 2018;169(7):467-473. doi:10.7326/M18-0850.

**Supplementary Table S1. Detailed case summaries of included reports**

This supporting-information table preserves the detailed case-level narrative extracted from the source manuscript. It should be uploaded as a separate supporting-information file.

Source-level appraisal. Because the evidence base is dominated by single case reports and small case series, a formal risk-of-bias instrument was not appropriate. Instead, each source was appraised narratively for study design (single case report, case series, or retrospective analysis), source type (peer-reviewed full text versus conference abstract), and completeness of reporting across three domains (baseline haemodynamics, complications, and follow-up/outcome). Of the 23 included publications, roughly half were conference abstracts with limited methodological detail, and reporting of baseline haemodynamics and device settings was frequently incomplete; findings should be interpreted in that light. Six patients reported across overlapping publications from the same centres were identified and counted once.

| **Study** | **Study context** | **Case summary** | **Indication / role of Impella-supported non-cardiac surgery** |
| --- | --- | --- | --- |
| Agrawal et al., 2024 | Case report; 2024; USA | A 30-year-old man with non-ischaemic cardiomyopathy, type 2 diabetes, hypertension, obstructive sleep apnoea, and morbid obesity (BMI 52) was admitted with decompensated heart failure and SCAI stage C cardiogenic shock. Initial management included milrinone and diuresis, but worsening symptoms necessitated VA ECMO support with concurrent Impella CP for LV unloading. ECMO was later weaned, and the Impella was upgraded to a 5.5 device via the axillary route. While on Impella 5.5 and dual inotropes, he underwent robotic sleeve gastrectomy. Three weeks postoperatively, he successfully received a HeartMate 3 LVAD. He was discharged 19 days later (BMI 39) and continued to lose weight, with a BMI of 34 at 3 months. He is planned for transplant listing 6 months after LVAD implantation. Overlaps with Sher et al. (2024, Case 1); counted once in the synthesis. | Sleeve gastrectomy as a bridge to advanced heart failure therapy (LVAD/transplant candidacy) in severe cardiogenic shock. |
| Alpuerto et al., 2022 | Case report; 2022; USA | A 37-year-old obese man with non-ischaemic cardiomyopathy and end-stage renal disease presented with worsening heart failure despite ongoing milrinone therapy. In preparation for dual organ transplantation, he underwent bilateral nephrectomy. Postoperatively, he became haemodynamically unstable and developed early signs of end-organ dysfunction despite intra-aortic balloon pump support and deteriorated to cardiac arrest during induction for escalation of support. The rationale for Impella use was emergent rescue of cardiogenic shock following major abdominal surgery and stabilisation to preserve candidacy for definitive transplantation. Following return of spontaneous circulation, Impella 5.5 was inserted emergently after return to theatre, providing flows up to 5.2 L/min and restoring haemodynamic stability. The device supported the patient through postoperative recovery while on continuous renal replacement therapy, with no reported device-related complications. He underwent successful combined heart and kidney transplantation 30 days later and remains well with good graft function at 5-month follow-up. | Rescue haemodynamic support following peri-induction cardiac arrest to enable recovery and bridge to heart–kidney transplantation. |
| Anderson et al., 2024 | Case report; 2024; USA | A 66-year-old woman with multiple comorbidities presented with abdominal pain, bloody diarrhoea, weight loss, and a fall. She developed refractory shock requiring multiple vasopressors and was found to have severe LV dysfunction, and CT showed significant pneumoperitoneum indicating colonic perforation secondary to an obstructing sigmoid malignancy. To stabilise her for surgery, she was supported with Impella 5.5 using a heparin-free purge. After recovery, her LV function normalized, and she was discharged after rehabilitation. | Haemodynamic stabilisation for emergency colorectal surgery in refractory shock with severe LV dysfunction. |
| Gopalan et al., 2022 | Case report; 2022; USA | 60-year-old male with end-stage heart failure (ESHF) and BMI of 41, undergoing workup for cardiac transplant but requiring bariatric surgery. LVEF 20-25%. Placed on Impella CP prior to sleeve gastrectomy. Impella supported for 36 hours post-op, and the patient was discharged 4 days later. | Sleeve gastrectomy to enable weight loss and heart transplant candidacy in end-stage HF. |
| Atoui et al., 2010 | Case report; 2010; Canada | A 56-year-old man with end-stage ischaemic cardiomyopathy (LVEF 15%) awaiting cardiac transplantation developed acute cholecystitis refractory to medical management and cholecystostomy drainage, with ongoing biliary sepsis and recurrent tube complications. Given the need for definitive source control and the prohibitive cardiac risk of surgery, a decision was made to proceed with laparoscopic cholecystectomy under mechanical circulatory support. Impella LP 2.5 was inserted pre-operatively via the femoral artery in the catheterisation laboratory to provide haemodynamic support during anaesthesia and pneumoperitoneum, which were anticipated to significantly compromise cardiac output. During surgery, induction of anaesthesia and CO₂ insufflation led to a marked drop in cardiac index, which was only maintained above 2.0 L/min/m² with near-maximal Impella flow (2.1–2.3 L/min). Despite these physiological stresses, the patient remained haemodynamically stable with minimal vasopressor support. The procedure was completed laparoscopically, and the device was explanted immediately postoperatively. The postoperative course was uncomplicated, with early extubation and discharge on postoperative day three. The authors conclude that the Impella device offers a promising alternative to intra-aortic balloon pump (IABP) support in high-risk non-cardiac surgeries by providing superior ventricular unloading and cardiac output augmentation. | Perioperative haemodynamic support to enable laparoscopic cholecystectomy in severe cardiomyopathy awaiting transplant. |
| Bhansali et al., 2020 | Case report; 2020; USA | A 65-year-old man with advanced ischaemic cardiomyopathy (EF 15%), ventricular tachycardia, and inotrope-dependent heart failure was being evaluated for advanced therapies. Work-up identified an adrenal mass consistent with a catecholamine-secreting tumour (pheochromocytoma/paraganglioma), which precluded progression to LVAD or transplantation. He was initiated on pre-operative alpha-blockade followed by beta-blockade for catecholamine control, but subsequently deteriorated into refractory cardiogenic shock despite escalating inotropes and intra-aortic balloon pump support. Impella 5.0 was inserted as a bridge-to-decision strategy to stabilise haemodynamics and end-organ function. Following optimisation and multidisciplinary discussion, the rationale for proceeding to surgery was to remove the tumour as a reversible barrier to definitive heart failure therapy. The patient underwent open abdominal tumour resection (adrenalectomy with right nephrectomy) under ongoing Impella support. The procedure was completed successfully, with resection of a 4 cm mass adherent to the renal vessels. Histology confirmed paraganglioma with clear margins and no vascular invasion. Postoperatively, the patient improved and was subsequently deemed eligible for advanced therapies, undergoing implantation of a durable HeartMate 3 LVAD. | Haemodynamic support for adrenalectomy in pheochromocytoma crisis as a bridge to LVAD candidacy. |
| Bravim et al., 2009 | Case report; 2009; Canada | A 55-year-old man with end-stage ischaemic cardiomyopathy (LVEF 15%), listed for heart transplantation (ASA IV), was scheduled for elective laparoscopic cholecystectomy following recurrent biliary symptoms despite prior cholecystostomy. His comorbidities included left ventricular thrombus, congestive hepatopathy, and prior gastrointestinal bleeding. Given his severely limited cardiac reserve and the anticipated haemodynamic stress of anaesthesia, pneumoperitoneum, and reverse Trendelenburg positioning, the rationale for mechanical circulatory support was to mitigate intraoperative reductions in cardiac output and maintain coronary and end-organ perfusion. Impella 2.5 was inserted pre-operatively in the catheterisation laboratory on the day of surgery, under light sedation, with invasive haemodynamic monitoring established (arterial line and pulmonary artery catheter). Anticoagulation was initiated with heparin (target ACT ~200 seconds). The device was set to deliver approximately 2.2 L/min of flow prior to transfer to theatre. The patient underwent laparoscopic cholecystectomy under general anaesthesia, with pneumoperitoneum pressures limited to 10 mmHg. Intraoperatively, haemodynamics remained stable with only low-dose vasopressor support, and there was no significant deterioration in cardiac index despite pneumoperitoneum and positioning. The procedure was completed uneventfully, anticoagulation was reversed, and the Impella device was removed immediately postoperatively. Postoperatively, the patient required brief ICU observation (24 hours). He experienced transient chest pain with mild troponin elevation but no ECG changes, which resolved with medical management. He was discharged home on postoperative day seven. Overlaps with the later full report by Atoui et al. (2010); counted once. | Perioperative support for  laparoscopic cholecystectomy |
| Brozzi et al., 2022 | Case series; 2022; USA | Patient 1: Obese patient with recurrent ventricular arrhythmia resulting in cardiogenic shock. Stabilised with IABP, diuretics, and milrinone. CP was then used (24 hours prior to surgery), followed by sleeve gastrectomy. Postoperatively, the patient recovered, lost 49 kg, and was listed for heart transplant.  Patient 2: Acute heart failure due to non-ischemic cardiomyopathy. Stabilised with IABP, diuretics, and IV milrinone. The patient declined LVAD due to unacceptable complications. 5.5 was implanted, and the patient underwent sleeve gastrectomy. Stable through surgery, lost 39 kg, and was listed for heart transplant. | Bariatric surgery (sleeve gastrectomy) in advanced heart failure to enable weight loss and heart transplant eligibility. |
| Dempsey et al., 2019 | Case report; 2019; UK | 33-year-old man presented with hypertensive crisis complicated by fulminant cardiogenic shock, severe left ventricular dysfunction, and hypoxaemic respiratory failure due to acute pulmonary oedema. CT aortography, initially performed to exclude aortic dissection, identified a necrotic enhancing adrenal mass consistent with phaeochromocytoma. He was commenced on VA-ECMO at the referring hospital and transferred to a specialist centre. Impella CP was inserted after transfer to unload an increasingly dilated left ventricle. Despite maximal medical therapy, he continued to experience recurrent hypertensive crises, and adrenalectomy was considered the only viable strategy to control catecholamine surges. Preoperatively, he received intravenous alpha- and beta-adrenoceptor blockade, fluid optimisation, and correction of glucose/electrolyte abnormalities. He underwent adrenalectomy while supported on dual MCS with VA-ECMO and Impella. Intraoperative management focused on avoiding catecholamine release, minimising tumour manipulation, and controlling blood pressure, heart rate, and arrhythmias. Following tumour removal, anticipated hypotension occurred and required noradrenaline, adrenaline, and vasopressin. Initial postoperative recovery was favourable, with myocardial improvement sufficient to consider ECMO explantation. However, despite cardiovascular stabilisation, he suffered a catastrophic intracranial haemorrhage and treatment was withdrawn. | Haemodynamic support for adrenalectomy in pheochromocytoma crisis with cardiogenic shock. |
| Dominedo et al., 2021 | Case report; 2021; Italy | A 28-year-old woman presented with hypertensive crisis complicated by pulmonary oedema, Takotsubo cardiomyopathy (LVEF 20–30%), and metabolic acidosis, progressing to refractory cardiogenic shock. She required urgent VA-ECMO with concomitant Impella support for circulatory stabilisation. Imaging subsequently revealed a 5 cm left adrenal mass consistent with pheochromocytoma, with the clinical course further complicated by acute kidney injury requiring renal replacement therapy and posterior reversible encephalopathy syndrome (PRES). The rationale for mechanical circulatory support was rescue of catecholamine-induced cardiogenic shock and preservation of end-organ function while establishing diagnosis and enabling definitive treatment. Following haemodynamic stabilisation and gradual recovery of ventricular function, ECMO and Impella were weaned and removed. The patient was then medically optimised with alpha-blockade (urapidil, doxazosin) followed by beta-blockade, achieving blood pressure control and preparing for surgery. After stabilisation, she underwent laparoscopic adrenalectomy approximately 7–10 days after initiation of alpha-blockade, with the aim of removing the source of catecholamine excess. Postoperatively, the patient demonstrated complete recovery of left ventricular function (LVEF 58%), resolution of neurological deficits, and was discharged without residual complications. | Stabilisation of catecholamine-induced cardiogenic shock (phaeochromocytoma) enabling delayed adrenalectomy. |
| Farhat et al., 2008 | Case report; 2008; France | A 50-year-old male with a background of end stage heart failure, smoking, alcohol addiction and dyslipidaemia was referred for pre-transplant assessment. He was found to have multi vessel stenosis with necrotic anterior and inferior wall on MRI with LVEF 18%. An ICD was inserted. Systematic body scan revealed an infra-renal aortic aneurysm extending to the iliac bifurcation. A decision was made to proceed to surgery with impella support. Impella LP 5.0 was inserted intra-operatively via the right subclavian artery (through a Dacron graft) after induction of general anaesthesia, and advanced across the aortic valve under fluoroscopic guidance. The device provided flows of approximately 4–4.5 L/min throughout the procedure. The patient underwent open abdominal aortic aneurysm repair with Dacron graft replacement. During the operation, a transient episode of ventricular fibrillation occurred but resolved spontaneously, with haemodynamic stability maintained by Impella support. The device was weaned and removed at the end of the procedure without the need for inotropic support. Postoperatively, the patient was extubated early and had no cardiac complications. His course was complicated by urinary catheter-related infection and septic thrombophlebitis requiring treatment, but he was ultimately discharged home on postoperative day 21. | Haemodynamic support for open AAA repair in severe HF during transplant work-up. |
| Gieseke et al., 2024 | Retrospective analysis; 2024; USA | Retrospective analysis of 24 patients undergoing laparoscopic cholecystectomy due to gallstone/ischaemic cholecystitis. Within this cohort six patients (25%) received Impella support as part of a broader mechanical circulatory support strategy. Impella was used both as a standalone device and in combination with other modalities, including extracorporeal membrane oxygenation and left ventricular assist devices. Surgery was typically delayed (mean 15 days from diagnosis) to allow stabilisation. All patients underwent laparoscopic cholecystectomy under cardiac anaesthetic supervision, with low-pressure pneumoperitoneum (10 mmHg), invasive haemodynamic monitoring, and tailored use of inotropes and circulatory support. Timing of impella insertion and removal is not clearly specified. While laparoscopic surgery was technically feasible in all cases, with no conversions to open, outcomes in the overall cohort were characterised by high complication rates, including bleeding requiring transfusion (53%), reoperation (37.5%), and reintervention (71%). Among the 21 reoperations reported, the majority were related to cardiac device management and critical care complications, including Impella removal or exchange, escalation to LVAD or ECMO, and mediastinal re-exploration for bleeding. Only one reoperation (4%) was attributable to the index cholecystectomy and did not represent a true surgical complication. Overall 30-day mortality was 20.8% in the cohort and was attributed to underlying cardiac disease and multisystem organ failure. Given the small number of Impella-supported patients and the heterogeneity of concomitant mechanical circulatory support, outcomes specific to Impella cannot be isolated. | Laparoscopic cholecystectomy for biliary sepsis (calculous/acalculous) in cardiogenic shock or advanced HF. |
| Goswami et al., 2019 | Case report; 2019; USA | 68-year-old female with end-stage liver disease secondary to autoimmune hepatitis and hepatitis C (MELD 34), chronic kidney disease, and hepatorenal syndrome. Medical history includes hypertension, COPD, anaemia, RCC, smoking, alcohol use, and cocaine use. She had two previous admissions for decompensated liver disease. Initially sent to the operating theatre for liver transplantation, 8L of ascites was drained. However, she developed increasing vasopressor requirements (maximal doses of norepinephrine 0.5 μg/kg/min, epinephrine 0.2 μg/kg/min, and vasopressin 0.12 U/kg/min). Liver transplantation was completed, but renal transplant was deferred. Bedside TTE showed a dilated LV with EF 10%, consistent with Takotsubo cardiomyopathy. A decision was made to proceed with renal transplant on VAECMO/Impella support. The rationale for mechanical circulatory support was rescue of severe, potentially reversible cardiogenic shock and preservation of end-organ function, while allowing completion of a time-critical transplant procedure (to avoid loss of a donor organ). VA-ECMO was initiated with concomitant Impella insertion (ECMELLA) on the day following liver transplantation, providing both systemic perfusion and left ventricular unloading. Under this support, the patient underwent renal transplantation, which was considered time-sensitive due to prolonged cold ischaemia of the donor kidney. The procedure was completed successfully, and the patient was managed postoperatively in the cardiothoracic ICU. With recovery of cardiac function, ECMO was weaned by postoperative day 6 and Impella approximately one week later. The patient was discharged after two months and ultimately returned home. | Support for time-sensitive staged renal transplantation after liver transplantation in acute severe biventricular/LV failure (Takotsubo-type physiology) to preserve candidacy and permit definitive transplant surgery. |
| Guarracini et al., 2024 | Case report; 2024; Italy | A 74-year-old man with advanced heart failure (LVEF 15%, NYHA IIIb) and multiple comorbidities (CKD, diabetes, prior CABG/PCI) was diagnosed with colorectal cancer. He demonstrated haemodynamic instability even during colonoscopy despite inotropic support, and was considered prohibitively high risk for surgery, with predicted mortality 15% and major complication risk 40%. The rationale for Impella use was to enable non-deferrable oncological surgery in a patient with severely compromised haemodynamics, where conventional anaesthesia and surgery would likely precipitate cardiovascular collapse, and where resection offered meaningful therapeutic benefit. Impella CP was inserted pre-operatively via the femoral artery under light sedation, prior to induction of general anaesthesia, to stabilise haemodynamics during the perioperative period. A bicarbonate-based purge solution was used to minimise bleeding risk. The patient underwent open left hemicolectomy with colostomy under Impella support, with device flow set at approximately 3.5 L/min. Full support was required at key physiological stress points (induction, positioning, and end of procedure), demonstrating haemodynamic dependence. Intraoperative blood loss was minimal (100 mL), and haemodynamics remained stable throughout the procedure (duration 140 minutes). Impella was weaned and removed at the end of the procedure (10 mins prior to extubation), with short-term postoperative inotropic support. The postoperative course was uneventful, and the patient was discharged home after 7 days. At 6 months follow-up, he remained alive with stable haemodynamics. This patient is included within the Guarracini et al. (2026) cohort; counted once. | Non-deferrable colorectal cancer resection in severe HF with haemodynamic instability. |
| He et al., 2024 | Case report; 2024; USA | 51-year-old female with a background of CAD, CKD3, diabetes, and hepatitis C cirrhosis complicated by ascites, varices, portal hypertension, and hepatic encephalopathy. During liver transplant assessment, she was found to have 95% stenosis of the LAD/D1. MELD score 11. PCI to LAD/D1 was performed in June 2022, followed by hospitalization for fluid overload and diuresis. EF was 44%. Listed for heart-liver transplant, with MELD score 27. Started on plasmapheresis but developed reactive antibodies despite 5 rounds making candidacy for heart transplant difficult. After MDT discussion, a decision was made for liver-kidney transplant with Impella support and delisted for heart transplant. Impella 5.5 was inserted intra-operatively via the right axillary artery at the time of transplant surgery, prior to or at induction, and maintained at a support level of P7 throughout the procedure. The patient underwent combined liver–kidney transplantation under Impella support, which was completed uneventfully. Postoperatively, she was extubated the following day and gradually weaned from vasopressors and device support. Impella was weaned and removed 5 days postoperatively, after haemodynamic stabilisation. The patient was discharged to rehabilitation 15 days post-transplant and subsequently home after further recovery. | Combined liver/kidney transplantation in a high-risk patient with cardiac dysfunction. |
| Lee et al., 2023 | Case report; 2023; USA | A 58-year-old man presented with new-onset severe heart failure (EF 22%) and cardiogenic shock (cardiac index 1.4 L/min/m²) requiring inotropic support and Impella, escalated from CP to 5. Further evaluation identified a renal mass suspicious for renal cell carcinoma. As the malignancy precluded progression to advanced heart failure therapies (e.g. transplantation or durable LVAD), and therefore represented a potentially reversible barrier to definitive treatment, a multidisciplinary decision was made to proceed with surgical resection. He underwent robotic-assisted partial nephrectomy under Impella support, with intraoperative flow increased from P3 to P5, and tolerated the procedure without reported haemodynamic compromise. | Partial nephrectomy for renal malignancy in cardiogenic shock/severe HF. |
| Montisci et al., 2020 | Case report; 2020; Italy | A 58-year-old man with anterior ST-elevation myocardial infarction complicated by refractory cardiogenic shock (LVEF ~25%) was initially supported with IABP, then escalated to Impella CP, and subsequently to Impella 5.0 via axillary access due to persistent heart failure and lack of recovery. Despite haemodynamic stabilisation and end-organ recovery, repeated attempts to wean from Impella failed. Further evaluation identified a rectal adenocarcinoma, which precluded candidacy for heart transplantation. The rationale for surgery was to remove the malignancy as a prerequisite for consideration of definitive heart failure therapy (LVAD/transplant) in a patient who remained dependent on mechanical circulatory support. The patient underwent low anterior resection with total mesorectal excision and diverting loop ileostomy while on ongoing Impella 5.0 support. Anticoagulation (bivalirudin) and antiplatelet therapy were carefully managed perioperatively. The intraoperative course was uneventful, with acceptable blood loss (<1000 mL) and no device-related complications. Postoperatively, the patient demonstrated recovery with mobilisation and enteral feeding, and later underwent ileostomy closure. However, due to prolonged mechanical support and ICU stay, he developed multidrug-resistant Klebsiella pneumoniae infection and died after 61 days of Impella support, before planned LVAD implantation. | Low anterior resection for colorectal adenocarcinoma as a bridge to attempted later LVAD strategy. |
| Pietrasik et al., 2024 | Case report; 2024; Poland | 47-year-old man with Marfan syndrome, chronic kidney disease and history of ascending aortic aneurysm with aortic root repair and postoperative MI presented with a complex thoracoabdominal aortic dissection. Given his high surgical risk (EuroScore II 32%) and history of cardiac complications, the Heart Team performed repeat endovascular aortic repair (EVAR) with Impella CP support. Impella CP was inserted pre-operatively via right axillary artery cutdown, providing flows sufficient to augment cardiac output (reported up to ~7.9 L/min). The Impella device provided stable haemodynamics during the procedure. Impella was removed after approximately 4.5 hours at the end of the procedure, and the patient had an uncomplicated postoperative course, requiring only a short ICU stay (2 days) and discharge home by day 9. | Endovascular repair of thoracoabdominal aortic dissection in a patient with very high cardiac operative risk. |
| Shah et al., 2021 | Case report - Abstract; 2021; USA | A 66-year-old man with decompensated alcoholic cirrhosis underwent orthotopic liver transplantation with normal preoperative cardiac function (EF ~65%). Intraoperatively, he developed acute biventricular failure immediately post-reperfusion, with EF falling to 10–16% and severe haemodynamic instability requiring high-dose inotropes and vasopressors. The rationale for mechanical circulatory support was rescue of acute perioperative cardiogenic shock and preservation of the newly transplanted liver graft, in the setting of sudden biventricular failure. The patient was initially placed on VA-ECMO for immediate stabilisation, followed by escalation to biventricular mechanical support (Impella 5.5 for LV support via axillary access and Protek-Duo RVAD) to provide effective ventricular unloading and systemic perfusion. The patient was managed in the ICU with ongoing mechanical support. Over the following days, cardiac function progressively recovered, allowing weaning from vasopressors and extubation. The RVAD was removed on postoperative day 8 and Impella on day 13, with restoration of left ventricular function (EF 52%). Liver graft function was preserved throughout. | Rescue support after orthotopic liver transplantation for acute biventricular failure, to preserve graft function and enable recovery. |
| Sher et al., 2024 | Case Series (3 patients); 2024 | Case 1: A 30-year-old man with advanced non-ischaemic cardiomyopathy (LVEF 14%), class IV obesity (BMI 51.9 kg/m²), and type 2 diabetes presented with cardiogenic and septic shock. He required VA-ECMO with Impella CP for left ventricular unloading and was subsequently upgraded to Impella 5.5 on hospital day 11 for ongoing support. After haemodynamic stabilisation, he underwent laparoscopic sleeve gastrectomy on hospital day 26 under Impella support. The procedure was completed successfully without intraoperative complications. Postoperatively, he remained on Impella support with improving haemodynamics and reduced inotropic requirements. The device was removed on hospital day 50, followed by successful LVAD implantation, and he was discharged on hospital day 68. Case 2: A 64-year-old man with advanced non-ischaemic cardiomyopathy (LVEF 15–20%), severe mitral regurgitation, and class III obesity (BMI 41.9 kg/m²) presented with cardiogenic shock. Despite optimisation with inotropes, he remained haemodynamically unstable and underwent Impella 5.5 implantation on hospital day 16 via axillary access. After a period of stabilisation, laparoscopic sleeve gastrectomy was performed on hospital day 26 under Impella support. The procedure was completed without complication. He remained supported postoperatively and subsequently underwent successful LVAD implantation on hospital day 33, with discharge on hospital day 56. Case 3: 26-year-old man with dilated cardiomyopathy (LVEF 15–20%) and class IV obesity (BMI 52.7 kg/m²) presented with acute decompensated heart failure. Following initial medical stabilisation, Impella 5.5 was implanted on hospital day 39. Laparoscopic sleeve gastrectomy was performed the following day under Impella support. The procedure was completed successfully. His postoperative course was complicated by hepatic and pulmonary abscesses, which resolved with antibiotic therapy. He remained on Impella support before undergoing LVAD implantation on hospital day 79 and was discharged on hospital day 110. | Sleeve gastrectomy as a bridge to durable LVAD and/or transplantation in advanced heart failure with severe obesity |
| Tagliafierro et al., 2023 | Case report - Abstract; 2023; USA | A 74-year-old man with severe multivessel coronary artery disease (including a critical left main lesion) presented with acute coronary syndrome and was planned for urgent coronary artery bypass grafting. However, staging imaging revealed a haemorrhagic intracranial metastasis (melanoma), creating a competing priority and rendering immediate cardiac surgery unsafe. The rationale for Impella use was to provide temporary haemodynamic support to enable urgent neurosurgical resection of a bleeding intracranial metastasis, thereby allowing subsequent definitive cardiac revascularisation in a patient with critical coronary disease. Impella 5.5 was inserted pre-operatively as temporary mechanical circulatory support, particularly important given the need to avoid standard anticoagulation strategies in the context of active intracranial bleeding (a bicarbonate purge solution was used as an alternative). The patient underwent craniotomy and successful resection of the haemorrhagic brain metastasis under Impella support, with no further intracranial bleeding despite subsequent anticoagulation. Following stabilisation, he proceeded to successful multivessel CABG one week later, after which the Impella device was explanted. Postoperatively, he recovered well and was extubated early, with plans for adjuvant oncological therapy. | Urgent craniotomy/resection of haemorrhagic brain metastasis in a patient with critical coronary disease requiring stabilisation before later CABG. |
| Guarracini et al., 2026 | Retrospective case series; 2026; Italy | Single-centre cohort of 10 patients with advanced heart failure (LVEF ≤35%) undergoing high-risk non-cardiac surgery with preoperative Impella CP support. Procedures included gastrointestinal (n=5; hemicolectomy, colorectal surgery, gastric bypass), orthopaedic (n=4; joint replacement/fixation), and vascular surgery (n=1; femoral–popliteal bypass). The specific indications for colorectal procedures were not individually reported, although these likely included oncological resections or other non-deferrable gastrointestinal pathologies in high-risk patients. Impella CP (SmartAssist) was inserted pre-operatively in all patients via femoral access prior to induction of general anaesthesia, with flows typically set at ~3.5 L/min. Support was maintained throughout the procedure (mean duration 131 minutes), targeting stable mean arterial pressure and organ perfusion during key intraoperative stress phases. All procedures were completed successfully under Impella support. The device was weaned and removed at the end of surgery (prior to extubation), with short-term postoperative inotropic support as required. Haemodynamic parameters improved perioperatively (e.g. MAP increased from ~67 to ~81 mmHg), and organ function was preserved. There were no major adverse cardiac events, no perioperative mortality, and no device malfunctions. Only one patient required transfusion. Median hospital stay was approximately 8 days, with sustained haemodynamic stability at follow-up (median 227 days). | Prophylactic perioperative haemodynamic support to enable high-risk non-cardiac surgery in patients with advanced heart failure and poor haemodynamic reserve. |
| Chandrasekhar et al., 2025 | Retrospective case series; 2025; USA | Three male patients (mean age 40.7 years) with advanced non-ischaemic cardiomyopathy and cardiogenic shock (mean BMI 48.3 kg/m²) were evaluated for advanced heart failure therapies but were ineligible for transplantation due to severe obesity (BMI >35). Initial management included inotropes or VA-ECMO (with Impella CP venting), but all failed to recover and were transitioned to Impella 5.5 via axillary access for ongoing temporary mechanical circulatory support. The rationale for Impella support was to enable bariatric surgery as a bridge strategy to restore eligibility for durable LVAD and eventual transplantation, given that obesity represented a reversible barrier to definitive therapy. Following haemodynamic stabilisation, all patients underwent sleeve gastrectomy under Impella 5.5 support, performed at a mean of 9.3 days after device implantation. Procedures were completed successfully. One patient developed an infection requiring prolonged antibiotics; otherwise, there were no major perioperative complications or bleeding events.  All patients subsequently underwent durable LVAD implantation without significant complications, although transient right ventricular dysfunction requiring inotropes was observed in all cases (one meeting INTERMACS criteria for early RV failure). No patient required RVAD support. At follow-up, there was 100% survival at 6 months and no 30-day readmissions, demonstrating successful transition from temporary support through bariatric surgery to durable mechanical support. The same three patients are reported by Sher et al. (2024); counted once. | Sleeve gastrectomy as a bridge to durable LVAD implantation |
